# Insulin Resistance Change and Antiretroviral Therapy Exposure in HIV-Infected and Uninfected Rwandan Women: A Longitudinal Analysis

**DOI:** 10.1371/journal.pone.0123936

**Published:** 2015-04-16

**Authors:** Eugene Mutimura, Donald R. Hoover, Qiuhu Shi, Jean Claude Dusingize, Jean D’Amour Sinayobye, Mardge Cohen, Kathryn Anastos

**Affiliations:** 1 Regional Alliance for Sustainable Development (RASD Rwanda) Kigali, Rwanda; 2 The State University of New Jersey, New Brunswick, New Jersey, United States of America; 3 School of Health Sciences and Practice, New York Medical College, New York, New York, United States of America; 4 Department of Medicine, Stroger Hospital, Chicago, Illinois, United States of America; 5 Albert Einstein College of Medicine and Montefiore Medical Center, Bronx, New York, United States of America; FIOCRUZ, BRAZIL

## Abstract

**Background:**

We longitudinally assessed predictors of insulin resistance (IR) change among HIV-uninfected and HIV-infected (ART-initiators and ART-non-initiators) Rwandan women.

**Methodology:**

HIV-infected (HIV+) and uninfected (HIV-) women provided demographic and clinical measures: age, body mass index (BMI) in Kg/(height in meters)^2^, Fat-Mass (FMI) and Fat-Free-Mass (FFMI) index, fasting serum glucose and insulin. Homeostasis Model Assessment (HOMA) was calculated to estimate IR change over time in log_10_ transformed HOMA measured at study enrollment or prior to ART initiation in 3 groups: HIV- (n = 194), HIV+ ART-non-initiators (n=95) and HIV+ ART-initiators (n=371). ANCOVA linear regression models of change in log_10_-HOMA were fit with all models included the first log_10_ HOMA as a predictor.

**Results:**

Mean±SD log_10_-HOMA was -0.18±0.39 at the 1^st^ and -0.21±0.41 at the 2^nd^ measure, with mean change of 0.03±0.44. In the final model (all women) BMI at 1^st^ HOMA measure (0.014; 95% CI=0.006-0.021 per kg/m^2^; p<0.001) and change in BMI from 1^st^ to 2^nd^ measure (0.024; 95% CI=0.013-0.035 per kg/m^2^; p<0.001) predicted HOMA change. When restricted to subjects with FMI measures, FMI at 1^st^ HOMA measure (0.020; 95% CI=0.010-0.030 per kg/m^2^; p<0.001) and change in FMI from 1^st^ to 2^nd^ measure (0.032; 95% CI=0.020-0.043 per kg/m^2^; p<0.0001) predicted change in HOMA. While ART use did not predict change in log_10_-HOMA, untreated HIV+ women had a significant decline in IR over time. Use or duration of AZT, d4T and EFV was not associated with HOMA change in HIV+ women.

**Conclusions:**

Baseline BMI and change in BMI, and in particular fat mass and change in fat mass predicted insulin resistance change over ~3 years in HIV-infected and uninfected Rwandan women. Exposure to specific ART (d4T, AZT, EFV) did not predict insulin resistance change in ART-treated HIV-infected Rwandan women.

## Introduction

Insulin resistance (IR) has been described in human immunodeficiency (HIV)-infected individuals treated with combination antiretroviral therapy (ART).[1–6] Development of IR and onset of diabetes in ART-treated HIV-infected adults raise concerns regarding type and duration of ART exposure.[3,4] Expanding use of ART in sub-Saharan Africa (SSA) has led to a notable decline in HIV-associated morbidity and mortality.[7,8] However, development of IR associated with use of ART in these patients could threaten their quality of life and well-being, as it is associated with inflammation and development of diabetes mellitus.[9] Thus the well-recognized benefits of ART treatment in HIV-infected African patients may potentially be accompanied by the development of IR, pre-diabetes and diabetes [2,4].

Most studies that have examined HIV- and ART-associated IR have been from industrialized countries, where ART regimens containing protease inhibitors (PIs) are often the backbone of treatment.[5,6] However, cumulative exposure to nucleoside reverse transcriptase inhibitor (NRTI) drugs, and in particular stavudine (d4T), may result in development of IR in both women and men with HIV infection from developed and developing countries.[1–4] Existing data in developing countries are conflicting and limited on the relationship of HIV infection itself with development of IR and metabolic derangements.[2,3,10] Data from industrialized countries suggest that IR and diabetes are more common in HIV-infected people and are risk factors for development of coronary heart disease, which exposes HIV-infected people to several other complications.[11]

Few studies have investigated the associations of HIV infection and ART use with IR in Africa.[2] Improved understanding of the relationship between IR and ART exposure in the context of cardiovascular disease and diabetes risk in HIV-infected Africans may inform and contribute to specific treatment guidelines and decisions.[12] In a cross-sectional analysis, we found that HIV infection itself, and HIV-related immunodeficiency were associated with greater insulin sensitivity.[13] We assessed IR change longitudinally in HIV-infected and uninfected Rwandan women.

## Methods

### Setting and Participants

Data were from the Rwanda Women’s Inter-association Study and Assessment (RWISA), a prospective observational cohort study that investigated the effectiveness and toxicity of ART in 710 antiretroviral naïve HIV–infected women using 226 uninfected women as controls. Eligible participants from HIV care clinics and grassroots associations of women living with HIV infection were enrolled in 2005. Participants provided written informed consent. The informed consent process included explanation of the study details, and a video describing the study, followed by group discussion, a question and answer period, and then a private standard written informed consent. Participants’ signed consent documents were locked up in an office. The encrypted database contained no personal identifying information. Informed consent documents were only accessible to the program director. This study was approved by the Rwandan National Ethics Committee (RNEC) and the Institutional Review Board (IRB) of Montefiore Medical Center, Bronx NY, USA. Inclusion criteria were ≥25 years of age, and if HIV-negative willingness to undergo voluntary counseling and testing for HIV at 6-month intervals, presence in Rwanda in 1994, and for HIV-infected women naïveté to ART, *except* possible exposure to single-dose nevirapine during pregnancy to prevent mother to infant HIV transmission.

At study enrollment and at 6-month intervals, participants provided information by interviewer-administered questionnaire including medical history, demographic information and disease characteristics. Physical examinations were performed and specimens taken for CD4+ cell count determination, full blood count, other laboratory studies and repositing for future assays. RWISA study details have been previously described.[14]

### Clinical Laboratory Measurements

Serum samples for the first measure of glucose and insulin for HIV-negative and HIV+ women not initiating ART were taken from enrollment specimens. Samples for women who initiated ART were taken from the last pre-ART visit within a year of ART initiation. CD4+ lymphocytes counts were determined at the Rwanda National Reference Laboratory, Kigali using a FACS counter (Becton and Dickinson, Immunocytometry Systems, San Jose, CA, USA). HIV infection was diagnosed by a testing algorithm, which required 2 positive results from commercial HIV-1 antibodies ELISA kits (HIV Vironostika, Netherlands, and Murex HIV-1.2, Oxford, UK). Using serum frozen at -80°C, insulin was measured with the Perkin Elmer alphaLISA human insulin kit on a Perkin Elmer Enspire Plate reader and glucose was measured using the Olympus America glucose hexokinase kit on an Olympus AU400 chemistry auto-analyzer, at the Albert Einstein College of Medicine, Bronx, NY. Of note, insulin and glucose tests were run on 17 different “batch” dates. Details of participants’ demographic and clinical characteristics regarding body composition, metabolic profiles and immunologic data have been previously reported.[13–15].

### Assessment of Change in IR Outcome

The outcome, insulin resistance, was estimated by the Homeostasis Model Assessment (HOMA), calculated as fasting insulin (μU/ml) × fasting glucose (mmol/L) ⁄ 22.5.[16] HOMA correlates well with insulin resistance measured by the gold standard euglycemic hyperinsulinemic clamp, and is a useful for large-scale assessment measure of IR in epidemiological studies.[17]

### Assessment of exposures

Exposure variables obtained at the first and second visits included age, waist to hip ratio (WHR) and body mass index (BMI) calculated as weight in kilograms divided by (height in meters)^2^. Fat mass index (FMI) and fat-free mass index (FFMI) were calculated from resistance and reactance using Kotler’s formulae [18] and standardized by height-squared for 85% of the women. For the previous exposures we calculated change from first visit (i.e., baseline) to second visit as the values at the second visit minus the value at the first visit. It should be noted that inclusion of FMI and FFMI into the models resulted in a sample size reduction of about 15% as some participants did not have these measures at both visits.

At each semi-annual follow-up visit participants were asked questions regarding exact date and names of specific type of ART used since the previous visit (verified by both clinical data and patients ART medication cards). Participants were then categorized into 1 of 3 groups depending on HIV status and self-reported ART use during this time period: (a.) HIV-infected ART-initiators, (b.) HIV-infected ART non-initiators and (c.) HIV-uninfected women. For those who initiated ART, duration of self-reported drug exposure was obtained for each ART regimen used up to the 2^nd^ HOMA measure. Proportions of HIV-infected ART initiators and the number of drugs that were reported within a given class of ART were also documented.

### Statistical Analysis

Both HOMA and insulin were skewed to the left and thus were Log base-10 transformed to achieve symmetry. While glucose was otherwise normally distributed, there was one outlier individual so observations above the 99^th^ percentile were Winsorized to this value. We compared change between two log_10_ transformed HOMA-IR measures separated in time by a median of 3.12 years in three groups of participants: HIV uninfected (n = 194), HIV-infected ART non-initiators (n = 95) and HIV-infected ART initiators (n = 371). As noted earlier, in ART initiators the first measure was taken prior to and the second measure taken after ART initiation. Linear regression models of these changes in log_10_-HOMA were fit using the HIV+ART use category (HIV-, HIV+-non-ART-initiator, HIV+-ART-initiator), BMI, WHR, FMI and FFMI at baseline, and change in BMI, WHR, FMI and FFMI from the baseline to second measure, and type and duration of ART usage as predictors. All models included the first log_10_ HOMA as a predictor. Two final predictive models were built using stepwise selection with HIV/ART-initiation status forced in and otherwise P to enter and P to stay equal to 0.10. The first excluded FMI and FFMI. The second included FMI and FFMI instead of BMI and thus excluded the 15% of subjects missing these data as described earlier.

## Results

### Participant Characteristics

**Fig 1:**
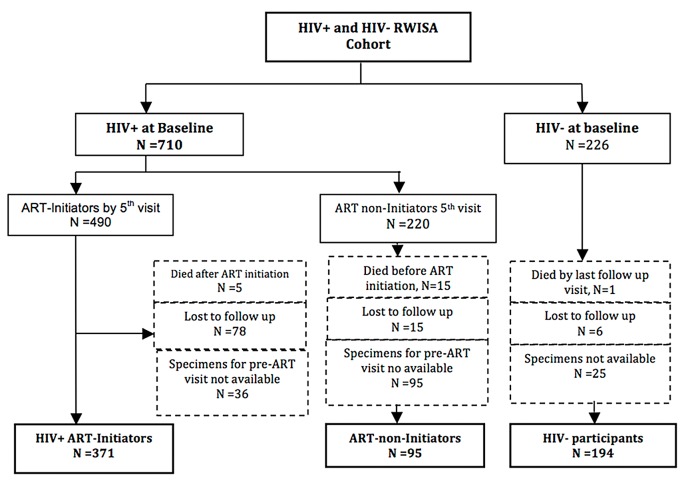
Flow Diagram RWISA longitudinal insulin resistance study; RWISA, Rwanda Women’s Interassociation Study Assessment; HIV, Human immunodeficiency virus; ART, antiretroviral therapy; HIV+, HIV-infected; HIV-, HIV-uninfected.

As shown in Fig 1, of the 710 HIV+ participants at baseline, 490 (69.0%) initiated ART by 5^th^ visit. Of these 490 participants, 5 died, 78 were lost to follow up (LTFU) and 36 had no available specimens leaving a total of 371 HIV+ ART initiators. Of the 710 HIV+ participants, 220 were non- ART initiators, of whom 15 died, 15 were LTFU and 95 had no specimens available leaving a total of 95 ART non-initiators. Of the 226 HIV uninfected participants, 1 died, 6 were LTFU and 25 had no available specimens leaving a total of 194 HIV- participants. Thus, a total of 660 women (194 HIV-uninfected, 95 HIV-infected ART non-initiators and 371 HIV-infected ART initiators) were included in this longitudinal analysis. FMI and FFMI at both HOMA measures was not available from 97 (15%) of the final women (Fig 1).

The HIV-uninfected women (HIV-) were older [mean age 42.3 vs. 33.6 (HIV+ART-non-initiator) and 35.2 (HIV+ ART-initiator) years; p <0.001] with somewhat longer times from 1^st^ to 2^nd^ HOMA measure [2.91 vs. 2.58 (HIV+ART-non-initiator) and 2.72 (HIV+ ART initiator) years; p<0.001] (Table 1).

**Table 1:** Demographic and Clinical Characteristics of 660 RWISA Participants by HIV Status and ART Usage.

Characteristics	HIV Infection and HAART Use Group*			
	HIV-Uninfected Women, n = 194	HIV-Infected ART-Non-initiators, n = 95	HIV-Infected ART-Initiators, n = 371	P-value†
Age				
At HOMA 1^st^ Measure	42.3±10.5	33.6±6.2	35.2±7.1	<0.001
At 2^nd^ HOMA Measure	45.2±10.7	33.6±6.2	35.2±7.1	<0.001
Age Change	2.9±0.7	2.6±0.8	2.7±0.7	<0.001
Waist-to-Hip Ratio (WHR)				
At HOMA 1^st^ Measure	0.88±0.07	0.88±0.06	0.88±0.08	0.97
At 2^nd^ HOMA Measure	0.88±0.07	0.87±0.08	0.89±0.09	0.13
WHR Change	0.00±0.09	0.00±0.08	0.01±0.09	0.26
Body Mass Index (BMI)				
At HOMA 1^st^ Measure	21.2±3.8	22.1±4.2	21.3±3.9	0.16
At 2^nd^ HOMA Measure	22.2±4.3	22.8±4.7	22.3±3.9	0.56
BMI Change	1.00±2.33	0.77±2.19	0.95±3.01	0.79
Fat Mass Index				
At 1^st^ HOMA Measure	3.80±3.11	4.36±3.66	4.30±3.66	0.11
At 2^nd^ HOMA Measure	4.29±3.76	4.73±4.01	4.70±3.69	0.28
Fat Mass Index Change	0.61±2.57	0.63±2.07	0.51±2.86	0.88
Fat Free Mass Index (FFMI)				
At 1^st^ HOMA Measure	17.32±1.60	17.66±1.62	17.09±1.69	0.003
At 2^nd^ HOMA Measure	17.71±1.58	17.70±1.66	17.62±1.47	0.85
FFMI Change	0.31±1.54	0.02±1.44	0.51±1.63	0.011
	2.91±0.65	2.58±0.82	2.72±0.67	<0.001
HOMA				
1st Measure	1.19±1.10	1.15±1.03	0.84±1.02	<0.001
2^nd^ Measure	1.10±1.32	0.96±1.34	0.95±1.29	0.41
Change From 1^st^ to 2^nd^ HOMA Measure	-0.09±1.35	-0.19±1.27	0.10±1.38	0.09
Log_10_-HOMA**‡**				
1st Measure	-0.09±0.38	-0.10±0.39	-0.26±0.38	<0.001
2^nd^ Measure	-0.16±0.41	-0.23±0.41	-0.24±0.41	0.09
Log-_10_-HOMA Change§	-0.07±0.42	-0.14±0.41	0.02±0.45	0.002
Log_10_-Insulin, mIU/L				
1st Measure	0.62±0.37	0.62±0.37	0.48±0.37	<.001
2^nd^ Measure	0.57±0.39	0.49±0.39	0.48±0.39	0.029
Log-_10_-Insulin Change	-0.05±0.40	-0.13±0.37	0.00±0.42	0.016
Glucose, gms/dl				
1^st^ Measure	80.3±9.6	78.9±9.85	75.5±9.8	<0.001
2^nd^ Measure	76.0±10.8	77.2±10.3	78.0±9.2	0.08
Glucose Change	-4.3±11.4	-1.7±11.4	2.5±12.1	<0.001

*Data are presented as Mean±SD;

**†**The p-value is from ANOVA;

**‡**Log_10_-HOMA is exponentiation of mean log_10_-HOMA;

§Change in Log_10_HOMA is exponentiation of mean change in log_10_-HOMA.

The HIV+ women who initiated ART had the lowest Log_10_-HOMA at 1^st^ measure [-0.26 vs. -0.10 (HIV+ART-non-initiator) and -0.09 (HIV-); p <0.001 comparing all three groups], as shown in Table 1. While the difference was not statistically significant, HIV+ women who initiated ART also had the lowest HOMA on the second measure. However, the Log_10_^-^HOMA change from the 1^st^ to 2^nd^ measure was highest for HIV+ ART initiators: +0.02 vs. -0.14 (HIV+ART-non-initiators) and -0.07 (HIV-), p = 0.002]. Similarly, the HIV+ women initiating ART had lower mean Log_10_-Insulin at 1^st^ insulin measure [0.48 vs. 0.62 (HIV+ ART non-initiators) and 0.62 (HIV-); p <0.001]. The HIV+ ART initiators were also more likely to have lower mean fasting glucose values at 1^st^ measure [75.5 vs. 78.9 (HIV+ ART-naïve) and 80.3 mg/dl (HIV-); p<0.001 for glucose.

### ART exposure

Table 2 presents ART exposure among the 371 HIV+ ART initiators. The HIV+ women who initiated ART were most likely by the 2^nd^ HOMA visit to have used stavudine (d4T), then zidovudine (AZT) and then efavirenz (EFV) (75.5 vs. 49.1% vs. 18.1%, p<0.001) with consequently longest mean reported duration of use of d4T then AZT then EFV (1.09 vs. 0.53 vs. 0.20 years; p<0.001).

**Table 2:** Description of ART usage among 371 women who initiated treatment.

Characteristic	Mean (±SD) or percentage
Years From 1^st^ HOMA Measure to ART Initiation	0.35±0.30
Years From ART Initiation to 2^nd^ HOMA Measure	2.38±0.74
Ever Reported Using Zidovudine	49.1%
Mean Years Reported Zidovudine Use	0.53±0.78
Ever Reported Using Stavudine	75.5%
Mean Years Reported Stavudine Use	1.09±0.92
Ever Reported Using Efavirenz	18.1%
Mean Years Reported Efavirenz Use	0.20±0.55

Proportions of HIV+ ART initiators: 26.7% used AZT, 62.5% used D4T, 89.1% used NRTI total and 1.3% used NRTI other than AZT or D4T.

### Predictors of change in insulin resistance

Table 3 presents unadjusted and adjusted predictors of change in log_10_-HOMA from 1^st^ to 2^nd^ measure using ANCOVA models. Most notably, after adjusting for the levels of log_10_-HOMA at the 1^st^ measure (which were lowest in HIV+ ART initiators) the change from the 1^st^ to 2^nd^ measures did not statistically differ by HIV status and treatment group (P = 0.19). In the first final stepwise linear predictive model among all participants that excluded FMI and FFMI as predictors, 1^st^ HOMA measure, BMI at 1^st^ HOMA measure (0.014; 95% CI = 0.006–0.021 per kg/m^2^; *p*<0.001) and change in BMI from 1^st^ to 2^nd^ measure (0.024; 95% CI = 0.013–0.035 per kg/m^2^; *p*<0.001) predicted insulin resistance (Table 3). In the second final linear predictive model among all women that included FFMI and FMI as predictors (instead of BMI and change in BMI), higher FMI at 1^st^ HOMA measure (0.020; 95% CI = 0.010–0.030 per kg/m^2^; *p*<0.001) and greater change in FMI from 1^st^ to 2^nd^ measure (0.032; 95% CI = 0.020–0.043 per kg/m^2^; *p*<0.001) predicted greater IR (Table 3). FFMI and change in FFMI did not predict change in HOMA.

**Table 3:** Unadjusted and adjusted predictors of change in Log_10-_HOMA from first to second measure from ANCOVA Models^1^,^2^.

Characteristics			Final Models^1^ ^,^ ^2^ ^,^ ^3^			
	Single Variable Baseline HOMA Adjusted Model^1^ ^,^ ^2^		Excludes Fat-Free and Fat Mass		Includes Fat-Free and Fat Mass	
**AMONG HIV-NEGATIVE AND POSITIVE SUBJECTS**	**Effect (95% CI)**	**p-value**	**Effect (95% CI)**	**p-value**	**Effect (95% CI)**	**p-value**
First HOMA Measure	-0.579 (-0.654, -0.505)	<0.001	-0.590 (-0.667, -0.514)	<0.001	-0.586 (-0.668, -0.504)	<0.001
Age (at 1^st^ HOMA Measure) years	-0.001 (-0.004, 0.002)	0.51	–	–	-	-
Age (at 2^nd^ HOMA Measure) years	-0.001 (-0.005, 0.002)	0.44				
Years: 1^st^ and 2^nd^ HOMA Measure	-0.032 (-0.074, 0.010)	0.14	-0.043 (-0.085, -0.001)	0.047	-0.043 (-0.085, -0.001)	0.091
HIV status and ART Use Group		0.19		0.09		0.09
HIV Negative (baseline)						
HIV Positive No-ART	-0.079 (-0.174, 0.015)		-0.100 (-0.194, -0.006)	0.037	-0.104 (-0.205, -0.003)	0.043
HIV Positive ART initiators	-0.002 (-0.070, 0.065)		-0.015 (-0.082, -0.052)	0.67	-0.007 (-0.078, 0.063)	0.84
WHR at 1^st^ HOMA Measure	0.241 (-0.171, -0.654)	0.25	–			
Change in WHR: 1^st^ to 2^nd^ Measure	0.037 (-0.402, 0.382)	0.84				
BMI at 1^st^ HOMA Measure	0.009 (0.002, 0.017)	0.015	0.014 (0.006, 0.021)	<0.001		
Change in BMI: 1^st^ to 2^nd^ Measure	0.018 (0.007, 0.029)	<0.001	0.024 (0.013, 0.035)	<0.001		
Fat Free-Mass at 1^st^ HOMA Measure	0.012 (-0.008, 0.033)	0.23				
Change in Fat Free-Mass: 1^st^ to 2^nd^ Measure	-0.018 (-0.038, 0.002)	0.071				
Fat Mass at 1^st^ HOMA Measure	0.011 (0.002, 0.021)	0.019			0.020 (0.010, 0.030)	<0.001
Change in Fat Mass 1^st^ to 2^nd^ Measure	0.028 (0.016, 0.040)	<0.001			0.032 (0.020, 0.043)	<0.001
**AMONG SUBJECTS WHO INITIATED ART**			–	–		
Ever Used Zidovuine	0.026 (-0.066, 0.118)	0.47				
Years Used Zidovudine	-0.029(-0.080, 0.023)	0.27				
Ever Used Stavudine	0.026 (-0.066, 0.118)	0.58				
Years Used Stavudine	0.023 (-0.020, 0.065)	0.29				
Ever Used Efavarenz	0.017 (-0.088, 0.121)	0.75				
Years Used Efavarenz	0.005 (-0.078, 0.069)	0.89				

Table presents Linear Effect Coefficient (Adjusted) and 95% Confidence Intervals.

The First Log_10_ HOMA Measure is included as a predictor in all Linear Models

No Final Model among Subjects who initiated ART as no characteristic was significant in single variable ANCOVA models.

In both of the final adjusted models, HIV serostatus and ART use independently predicted change in log_10_-HOMA (overall p = 0.037 and 0.043 respectively in the model with all women and the model restricted to those with FFMI and FMI). Neither ever use nor cumulative exposure to d4T, AZT or EFV predicted IR change among ART initiators.

## Discussion

In this longitudinal analysis of predictors of change in insulin resistance measured by log_10-_HOMA, baseline BMI and change in BMI, and in particular higher fat mass and greater change in fat mass strongly and independently predicted increase in insulin resistance from the 1^st^ to 2^nd^ measure in HIV-infected and uninfected African women after adjusting for the 1^st^ HOMA measure. We did not find increasing insulin resistance in HIV+ women compared to HIV- women. However, HIV+ women who did not initiate ART, and therefore experienced disease progression over the three years of the study, had an increase in insulin sensitivity—i.e. their IR declined significantly more (compared to HIV- women) from the 1^st^ to 2^nd^ visit, with greater decline associated with longer time between 1^st^ an 2^nd^ visit. Waist-to-hip ratio (WHR) and use of specific ART regimens did not predict change in insulin resistance in ANCOVA linear models.

Although the HIV-infected ART initiators had a small increase in HOMA in unadjusted analysis (and the other groups had a decrease in HOMA), in the final multivariate models that adjusted for time between visits and BMI or FMI, the significant difference among the HIV/treatment group was a greater adjusted *decline* in the HIV-infected women who did not initiate ART, with no significant difference between the ART initiators and the HIV-uninfected women. Although age is associated with increase in insulin resistance and decline in glucose tolerance, we did not observe any association between baseline and follow up age with change in IR, while change in age between visits had a borderline association (P = 0.058). We believe that subtracting out the 1^st^ HOMA measure in calculating the IR change accounts for any cumulative effects of age up to baseline and that the time difference between the two visits was too short to statistically be able to see the age effect.

We have previously investigated predictors of cross sectional insulin resistance in HIV-uninfected and HIV-infected antiretroviral-naïve Rwandan women, and found that HIV infection and advanced HIV disease were associated with increased insulin sensitivity.[13] Findings from this analysis inform our understanding of insulin resistance change over a time in treated and untreated HIV+ women. Even in the thinner individuals who make up this cohort, higher weight and weight increase (and specifically fat-mass) predicted higher risk for increased insulin resistance, while untreated HIV disease was associated with increasing insulin sensitivity. Our results are similar to those from previous studies in developed countries, showing associations of increased weight with insulin resistance and onset of diabetes in HIV-infected individuals.[19–24]

There exist few studies of IR change in HIV-infected sub-Saharan Africans. Two recent studies found a significant increase in IR in HIV-infected South Africans initiating ART.[10,25] Several reports from industrialized countries have found no association of HIV status with IR change or prevalence of IR or diabetes[21,24] while the evidence for IR change with ART use is conflicting, with some studies finding a positive association of IR with ART use while others do not.[22,26] Insulin resistance was not associated with HIV infection in a large North American cohort of HIV-infected and HIV-uninfected women, in which 48% of HIV-infected patients were on protease inhibitors (PI).[21] In a study to determine the prevalence and risk factors for diabetes in HIV-infected and uninfected individuals in the Veterans Cohort Study, Butt *et al*. observed that HIV infection was not associated with increased risk for diabetes, although use of NRTI therapy was.[22] Diabetes mellitus incidence in the Swiss Cohort Study was found to be higher with PI-containing but not NNRTI-containing ART regimens.[26] Cohorts from industrialized countries with higher BMI/FMI than in our study population have found that type and duration of ART use predicted insulin resistance.[5,6,21] However, our study participants had low baseline weight with mean BMI of ~21 Kg/m^2^, which is lower than in most HIV cohort studies of insulin resistance [6,21] and while fat mass measurement was not reported elsewhere, we suspect the same is true here.

We also found no associations between specific ART agents or duration of their use with insulin resistance among women who initiated ART. In a descriptive breakdown by ART type among the 371 women who initiated ART, the largest increases were seen among the d4T group, but these changes were too small to be statistically significant. Our study could not ascertain the effect of PI use, as the number of participants using PI-based regimens was too small to establish these relationships. Still these findings are consistent with a large cohort study in North America that assessed the frequency of pre-diabetes and insulin resistance in HIV-infected and uninfected women, and reported no association of pre-diabetes and insulin resistance with use of ART in a cross-sectional analysis.[21] While some studies from US and European countries have reported that exposure to combination ART[1,3,5,6,26] and in particular d4T and AZT, was associated with greater insulin resistance and onset of diabetes in ART-treated HIV-infected adults, most of these studies were of predominately white male populations and included protease inhibitor based ART regimens, whereas nearly all ART regimens reported here were NNRTI based used by African women. A report from South Africa found fasting glucose and HOMA scores increased significantly more among patients on d4T compared to tenofovir.[25] A cross-sectional study from India also reported higher risk for insulin resistance in patients treated with ART compared to ART-naïve HIV-infected patients.[2] By contrast, in one South African study Kiage *et al*. found that even short-term exposure to ART of 90 days after treatment initiation was associated with propensity towards development of insulin resistance regardless of the type of ART regimen used.[10]

Our previous cross-sectional analysis found no association of demographic factors such as education and occupation with cross sectional IR, and perhaps not surprisingly we observed none between demographic factors and IR change (data not shown) [13]. Although age is associated with increase in insulin resistance and decline in glucose tolerance, we did not observe a significant association of age with IR change, although change in age between visits had a borderline association (p = 0.058). We believe that subtracting out the 1^st^ HOMA measure in calculating the IR change accounts for any cumulative effects of age up to baseline, and that the time difference between the two visits was too little to statistically be able to see an age effect.

It is interesting that the change in glucose from 1^st^ to 2^nd^ visit was highest for HIV- infected ART initiators (+2.5 g/dL Vs. -1.7 for HIV-infected ART non-initiators and -4.3 for HIV-uninfected, p <0.001 (Table 1). This may be a Type-1 error or it may be capturing a more complicated aspect of insulin resistance pathogenesis that HOMA does not pick up. It does not transmit through to HOMA as insulin, which increases in the HIV-infected ART-initiators but decreases in the other groups, makes a much larger mathematical contribution to HOMA than does glucose. Also of note, the relatively large decrease in glucose among the untreated HIV+ women with a *decrease* in the insulin levels suggests that the lower glucose is the primary event and is not caused by a higher insulin.

We acknowledge some limitations to our analysis. First, this was not a randomized trial and the lower Log_10_-HOMA at the 1st measure among HIV-infected ART initiators complicates comparison of change from the 1^st^ to 2^nd^ measure in this group. However, this is likely the case in all other longitudinal studies of IR change after ART initiation. Secondly, our study lacked male participants, and it is unclear if our results can be generalized to HIV-infected men. Thirdly, we defined insulin resistance using HOMA, which is a surrogate marker and not the gold standard euglycemic insulin clamp technique.[16,17] However, HOMA values highly correlate with insulin clamp technique,[16] and have been shown to be a reasonable measure of insulin resistance in large epidemiologic studies.[27,28]. It should also be noted that there was high collinearity between a sample’s insulin/glucose testing (batch date) and the person’s HIV/ART use group leading to an unbalanced design on this characteristic. But batch date as a predictor was not statistically significant after HIV/ART risk group was adjusted for (data not shown).

Finally, An ANCOVA approach that included the first log_10_ HOMA as a predictor was used for linear regression. While ANCOVA models can be biased for estimating effects of subsequent treatments initiated, for example from regression to different means, models that do not include the baseline predictor are equivalently biased in the same settings and subject to higher variance.[29] This reflects regression to the mean type processes in observational studies that cannot be adjusted by any model and may in fact be partly responsible for the different patterns of association we see between adjusted and unadjusted associations of HIV-infection/ART use with insulin resistance. We believe, however, that as baseline CD4+ was the main selection criteria for being subsequently put on ART, and neither baseline CD4+ (nor pre-post baseline change in CD4+) had residual association with Log_10_HOMA change in adjusted models among HIV-infected women (data not shown), such biases are limited.

In summary, only baseline weight and change in weight and specifically fat mass were independently associated with an increase in insulin resistance over ~3 year period in HIV-infected and uninfected African women with low baseline levels of insulin resistance. HIV serostatus and antiretroviral therapy including cumulative exposure to d4T, AZT and EFV did not predict development of insulin resistance. Untreated HIV infection was associated with less insulin resistance over time. Since weight gain within the normal BMI range could increase the risk for developing insulin resistance, HIV-infected individuals (and others) of normal weight in developing countries should be warned against possible excessive weight gain. Participants in this study may have been protected by their thinness and young age but the effects of ART exposure may be magnified in settings where participants are heavier and older. Of particular note, in HIV-uninfected individuals, attainment of relatively modest weight stability goals is recommended, and would therefore be prudent for HIV-infected persons to do the same to avoid potential development of insulin resistance.

The effects of epidemiological transition in Africa from infectious disease morbidity to modern health risks of metabolic disease due to urbanization and lifestyle changes may influence the interplay between glucose regulation and ART exposure even in HIV-infected individuals with low body weight.[30] On the other hand, our finding of no increase in insulin resistance over time among HIV infected and uninfected persons in this study compared to other studies may be attributed to ancestry and sex differences between cohorts from the industrialized countries and African women in this cohort. It would likely be wise to encourage HIV-infected (as well as uninfected) individuals in developing countries to live a nutritionally healthy lifestyle to maintain normal body weight, which among other benefits may also mitigate the risk for development of insulin resistance. Further investigations of insulin resistance and/or glucose metabolism and regulation in HIV-infected and uninfected African individuals may be helpful. Additionally, future comparisons of predictors of insulin resistance change, type and duration of antiretroviral therapy and other non-HIV related factors are warranted in the developing world as underlying insulin resistance risk in the population is likely to increase.
